# Effects of Sex and Western Diet on Spatial Lipidomic Profiles for the Hippocampus, Cortex, and Corpus Callosum in Mice Using MALDI MSI

**DOI:** 10.1021/jasms.3c00446

**Published:** 2024-03-08

**Authors:** Catelynn C. Shafer, Jacopo Di Lucente, Ulises Ruiz Mendiola, Izumi Maezawa, Lee-Way Jin, Elizabeth K. Neumann

**Affiliations:** † Department of Chemistry, University of California, Davis. Davis, California 95616, United States; ‡ Department of Pathology and Laboratory Medicine, University of California Davis, Sacramento, California 95817, United States

## Abstract

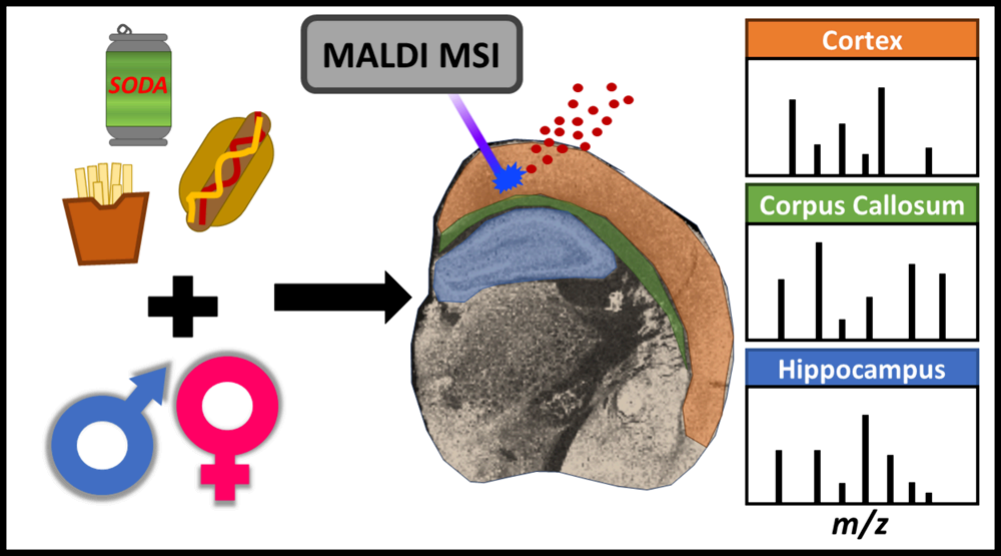

Diet is inextricably linked to human health and biological functionality. Reduced cognitive function among other health issues has been correlated with a western diet (WD) in mouse models, indicating that increases in neurodegeneration could be fueled in part by a poor diet. In this study, we use matrix-assisted laser desorption/ionization mass spectrometry imaging (MALDI MSI) to spatially map the lipidomic profiles of male and female mice that were fed a high-fat, high-sucrose WD for a period of 7 weeks. Our findings concluded that the cortex and corpus callosum showed significant lipid variation by WD in female mice, while there was little to no variation in the hippocampus, regardless of sex. On the other hand, lipid profiles were significantly affected by sex in all regions. Overall, 83 lipids were putatively identified in the mouse brain; among them, HexCer(40:1;O3) and PE(34:0) were found to have the largest statistical difference based on diet for female mice in the cortex and corpus callosum, respectively. Additional lipid changes are noted and can serve as a metric for understanding the brain’s metabolomic response to changes in diet, particularly as it relates to disease.

## Introduction

All aspects of human health depend on proper neurological function. Even slight disruptions in the metabolic profile of the brain can lead to negative changes in brain function effecting cognition, reasoning, memory, and hormone regulation.^1^ Genetic and environmental factors can affect the chemical environment of the brain, contributing to neurological disorders such as Alzheimer’s disease, Canavan, Parkinson’s disease, and many others.^2,3^ Thus, understanding the molecular microenvironment of the brain as it relates to changing external influences, such as diet, is crucial for potential disease prevention and therapeutic responses.^3^ One critical class of biomolecules contributing to neurological microenvironments is the lipidome.^4−6^ Lipids, which are crucial in cell membranes and signaling, are incredibly diverse and heterogeneous throughout the brain and must be maintained to regulate cell health and function.^7,8^ Lipids compose approximately half of the dry weight of the human brain with more than 100,000 lipid species, demonstrating their complexity and importance. Phospholipids, a critical lipid subclass comprising up to 60% of cell membranes, are composed of a polar headgroup with two hydrophobic side chains.^7,9,10^ These lipids are classified by their headgroup: phosphatidylcholine (PC), phosphatidylglycerol (PG), phosphatidylinositol (PI), phosphatidylethanolamine (PE), phosphatidic acid (PA), phosphatidylserine (PS), hexagonal ceramide (HexCer), and triglycerides (TG).^11^ Studies show that intake of a western diet (WD), containing excessive fat and sucrose, has numerous health concerns including obesity, psoriasiform dermatitis, and metabolic syndrome which contributes to increased risk of heart disease and stroke.^12,13^ Additionally, WD effects the neurological metabolic profile in mouse models, reduces neuroplasticity, contributes to cellular inflammation and microglial activation, and may be linked to neurodegenerative disease such as Alzheimer’s disease.^5,14−16^

While these studies show the importance of lipids in neurological function, demonstrating clear changes in the overall lipidomic profile as a function of diet, the methods used require homogenization of the sample and prevent spatial analysis. Spatial mapping of lipidomic changes remains critical to understanding disease onset and progression since brain regions are differentially affected in many neurological disorders.^17−19^ Studying lipid profiles using in situ mass spectrometry-based techniques are some of the most powerful methods to characterize lipid metabolism in tissues.^20−22^ There are several types of mass spectrometry imaging (MSI) instruments enabling spatial lipidomic analysis, including secondary ion mass spectrometry (SIMS), nanospray desorption electrospray ionization (nano-DESI) MSI and matrix-assisted laser desorption/ionization mass spectrometry imaging (MALDI) MSI.^22,23^ MALDI MSI has grown in popularity for neuroscience applications because it provides direct metabolomic information within cells/brain regions *in situ* and can measure thousands of lipids, proteins, peptides, and more while maintaining high mass accuracy and spatial resolution.^23−25^ MALDI MSI uses a UV laser to ionize and desorb molecules from the tissue surface while maintaining spatial distribution by rastering the laser across the tissue, resulting in ion images with a spatial resolution of ∼10 μm. Depending on tissue sample size, > 500,000 of multiplexed spectra containing hundreds or even thousands of ion species are collected, allowing for visualization of each ion’s relative abundance across the sample. This rich data set of spatially mapped molecular information has been used previously to study neurodegenerative diseases.^22,26,27^ Ion intensity comparisons can be made within a tissue to discriminate between regions or can be used to compare groups of tissues to show whether various factors, such as diet or sex, influence the molecular architecture of the tissue.

In this study, we use MALDI MSI to analyze lipidomic profiles of mouse brain samples as a function of sex and diet in specific brain regions, including cortex, corpus callosum, and hippocampus. In total, MALDI MSI enabled the detection and putative identification of 83 lipids from the PE, PI, and PS classes that are altered by diet and sex.

## Results

**Figure 1:**
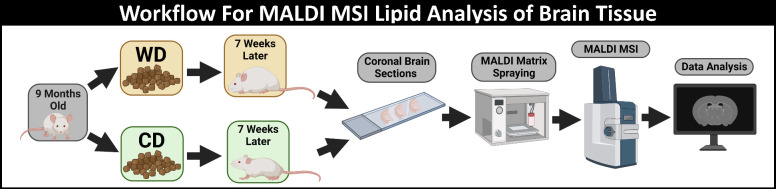
Overview of the experimental workflow. From left to right, workflow begins with 9-month-old mice fed either a WD or CD for 7 weeks, resulting in increased or normal weight gain, respectively. Cryosectioned tissues were thaw mounted onto ITO glass slides and subsequently coated with the MALDI matrix DAN. Data acquisition was performed on a MALDI mass spectrometer with data analysis completed through SCiLS software.

Obesity and related health effects continue to rise in western countries.^28^ This is largely due to the modern WD including foods low in nutrients, but high in fat and sugar, such as fast food.^29,30^ This study mimics these health affects in mice to show lipid variation in the brain as a function of diet by feeding wild type mice a WD replica, which induced significant weight gain in all mice (Figure 1). A standard MALDI MSI workflow was performed to obtain spatially resolved lipidomic data. WD studies began by treating nine month-old C57BL/6J wild type mice with a Western diet (WD; 42% kcal fat, 0.2% total cholesterol, and 34% sucrose by weight) or control diet (CD; 19.2% kcal fat, 0% added total cholesterol, and 12% sucrose by weight) (TD.88137 or TD.08485 respectively, Envigo, Indianapolis, IN)^5^ for 7 weeks. The murine brains were cryosectioned and sprayed with 1,5 diaminonaphthalene (DAN), and data acquisition was performed on a Bruker MALDI timsTOF fleX mass spectrometry system (Bruker Scientific, Billerica, MA). Data analysis and visualization was performed via SCiLS lab software (Bruker Scientific).

Negative ion mode was used for this experiment allowing for the detection of lipids that preferentially ionize with a negative charge; however, the same sample preparation allows for positive mode lipid detection without any additional steps other than selecting the appropriate instrument settings and would likely double the number of identifiable lipids. Indeed, due to differences in molecular structure, some lipids will only ionize in positive or negative ion mode but often not both. Thus, performing an MSI experiment in positive ion mode could then provide additional information that would supplement what is described herein. In total, 83 lipids were putatively identified and used in the comparative analysis (Tables S1 and S2). Data visualization enabled analysis of the cortex, hippocampus, and corpus callosum, since these brain regions are molecularly distinct.

**Figure 2:**
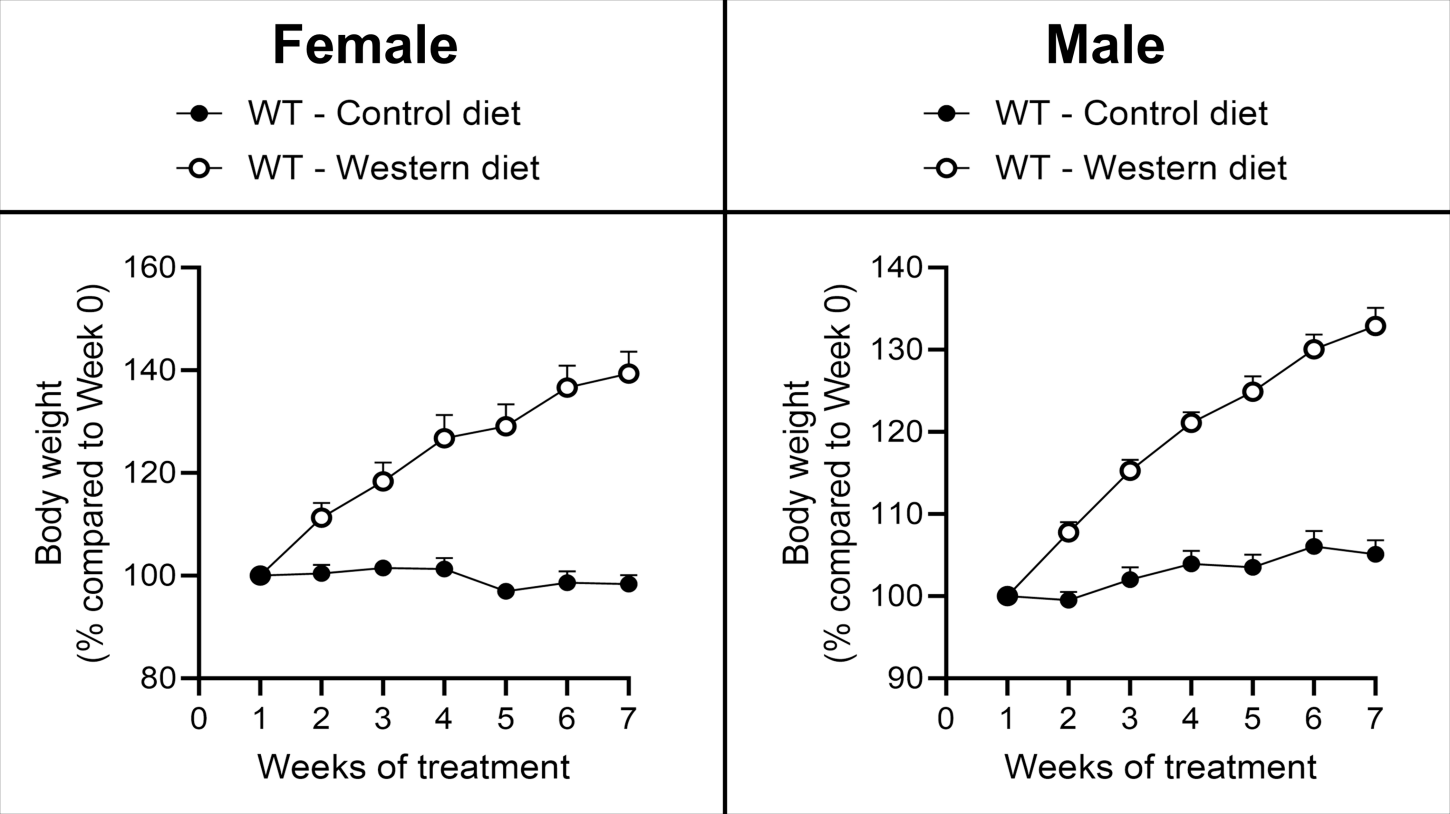
Weight gain for male and female mice fed WD and CD. Female and male mice gained 40% and 28% more weight, respectively, on WD in comparison to CD. *N* = 3 male and 3 female (total of six animals) per diet.

During the seven week period, the weight of WD-fed female and male mice increased 41% and 33%, respectively (Figure 2). In contrast, CD-fed female and male mice maintained a stable weight with 1% and 5% increase from week 0, respectively. While on CD, male mice gained more than females did; however, the reverse was true for WD, suggesting female mice may be more sensitive to diet-related weight changes. Due to the differences in weight gain as a function of sex and diet, we pursued analysis of neural tissue to determine sex and diet-based lipid variation in the brain. WD-fed mice experienced elevated weight gain in comparison to CD-fed mice, so we expected lipidomic differences to arise between the two groups, particularly given the known health complications from WD. In addition, sex is also expected to affect the lipid profile of the brain, because the inherent hormonal differences between male and female mice. Additionally, female mice gained more weight than male mice when fed a WD.

**Figure 3:**
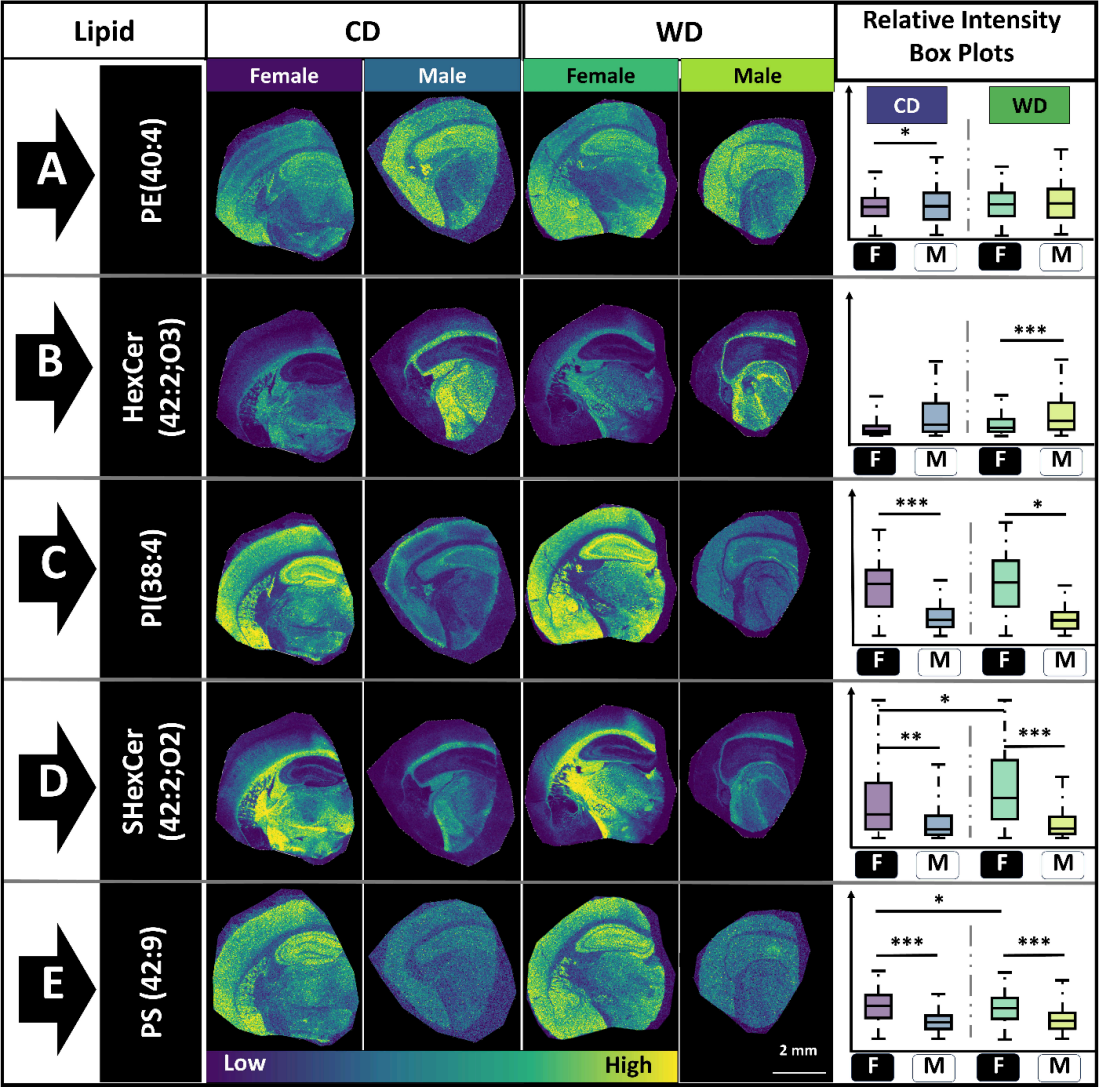
MALDI MS Images for five representative lipids; box plots show relative lipid intensity and statistical significance. Distribution and relative abundance of (A) PE(40:4), (B) HexCer(42:2;O3), (C) PI(38:4), (D) ShexCer(42:2;O2), and (E) PS(42:9) throughout the coronal section. MALDI MSI images are scaled from low abundance (dark blue) to high abundance (yellow). Stars indicate level of statistical significance; * = *p* < 0.1, ** = *P* < 0.05, *** = *P* < 0.01. *N* = 3 for all groups (Figures S1 and S2).

**Figure 4:**
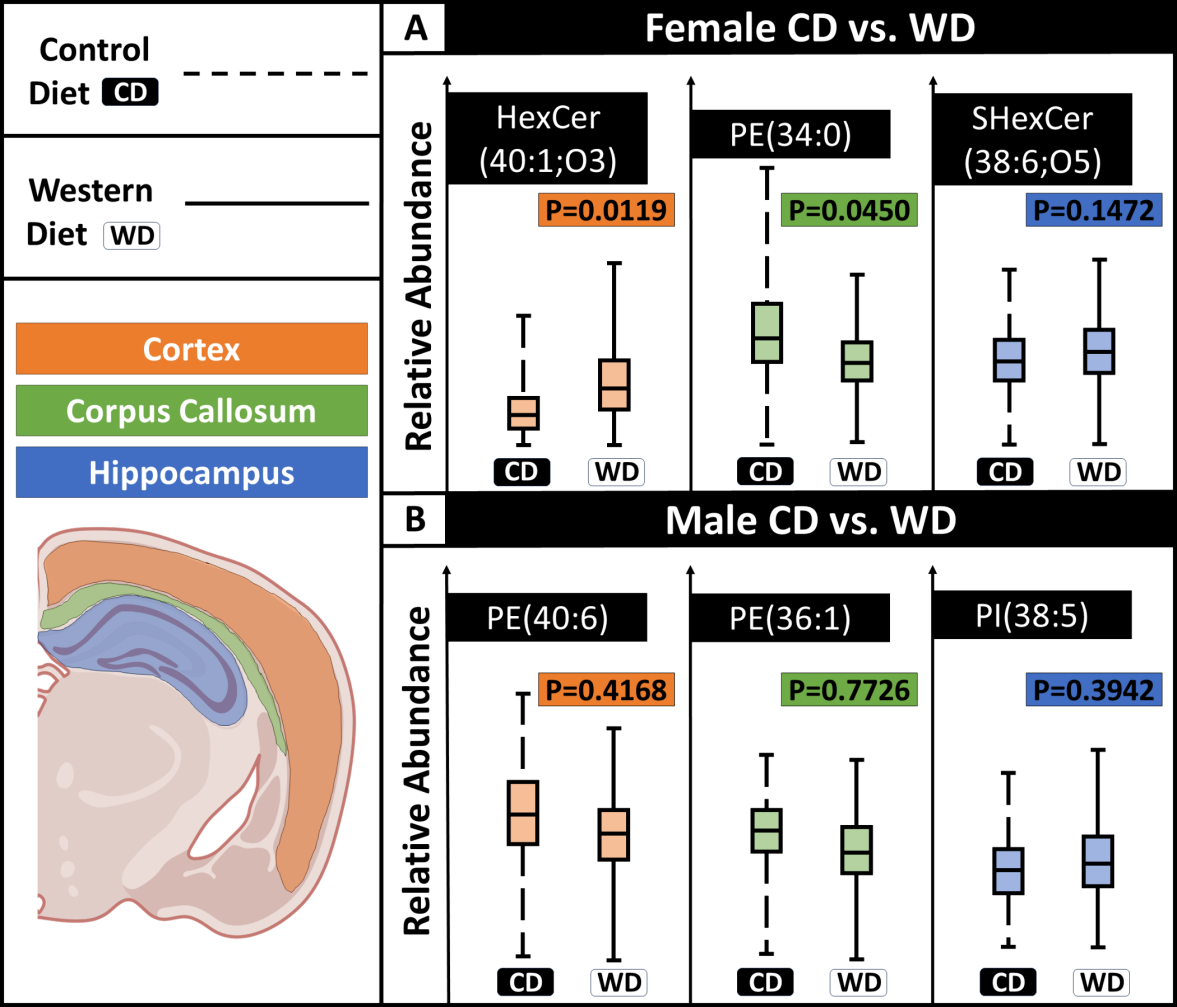
Variation in lipid abundance as a function of diet for different brain regions. The lipids selected have the highest ROC scores in the given brain region as a function of diet. (A) In female mice, HexCer(40:1;O3) and PE(34:0) show significant differences as a function of diet in the cortex and corpus callosum, respectively. Within the hippocampus, SHexCer(38:6;O5) is not statistically significant among the groups. (B) For male mice, none of the lipids were statistically significant. *N* = 3 male, 3 female per diet (six animals total).

Analyzing their lipidomic profiles shows that there are some lipids with no variation in their spatial distribution or abundance regardless of sex or diet, while others showed statistically significant variation as a function of sex and/or diet (Figure 3). Spatial lipid mapping revealed the relative abundance and distribution of the detected lipids within the sample, enabling lipid analysis of the whole brain much like LC-MS studies of bulk/homogenized tissue. Receiver operating curves (ROC) were used to determine the lipids that could effectively differentiate between different sexes and diets. The area under the curve of each ROC plot was adjusted to give a number between 0.5 and 1 with values closer to 0.5 representing poor discriminators, whereas values closer to 1 indicate perfect discriminators. Conventionally, values under 0.7 are considered poor discriminators, values between 0.7 and 0.8 are fair, values between 0.8 and 0.9 are good and values above 0.9 are excellent.^31^ ROC scores for all lipids were calculated to compare the effects of sex and diet on the lipid profiles of the whole brain, cortex, corpus callosum, and hippocampus (Table S2). As expected, values were obtained throughout the range of 0.5–1. Indeed, some lipids did not statistically change between the groups (e.g., PE(40:4), Figure 3A), while several did (Figure 3B–E). For example, PE (40:4) is expressed throughout the brain regardless of sex or diet, albeit with a higher abundance in the hippocampus and lateral segment of the cortex (Figure 3A). However, PS (42:9) shows a statistically significant variation as a function of sex (Figure 3 E). HexCer(42:2;O3) is localized to the corpus callosum and midbrain region and is significantly more abundant in the WD fed male mice compared to their female counterparts (Figure 3B). Alternatively, PI(38:4) is expressed within the hippocampus and is statistically different between male and female mice for both CD and WD fed samples (Figure 3C). In contrast, both HexCer(42:2;O3) and PI(38:4) are statistically different between male and female mice but are not statistically different based on diet (Figure 3B,C). Overall, the five representative lipids show higher variation as a function of sex than diet, with two lipids varying little between the groups. Of the five lipids, PS(42:9) and SHexCer (42:2;O2) show low statistical significance in female mice as a function of diet. When considering the whole brain, eight lipids with the highest ROC (<0.67) showed no statistical significance (Table S3). PS(42:9) and ShexCer (42:2;O2) showed statistical significance only in female comparisons. The low number of statistically significant lipids could partially be due to region-specific variation, where lipids vary more by diet in subregions than was observed over the whole brain (Figure 4). A longer time frame of WD feeding may be required to observe more significant differences in the lipidome. Our lipidomic data showed that PI(38:4) was significantly different between the sexes, suggesting enhanced signaling within female mice brains. Additionally, PE(40:4) was distributed all over the brain at similar abundances, regardless of sex or diet, indicating that PE(40:4) may be important for regulation of housekeeping functions. Overall, numerous PE and PS lipids were detected and are crucial membrane lipids whose breakdown has been observed as a metabolic defect for Alzheimer’s disease and epilepsy.^32,33^ Differences in the lipidomic profile may or may not indicate negative health outcomes, as some differences may be protective responses to environmental changes, including diet. For instance, some heat shock proteins are known to interact with lipids in response to cellular stress and do not inherently indicate that lipid changes are directly related to functional differences.^34,35^

**Figure 5:**
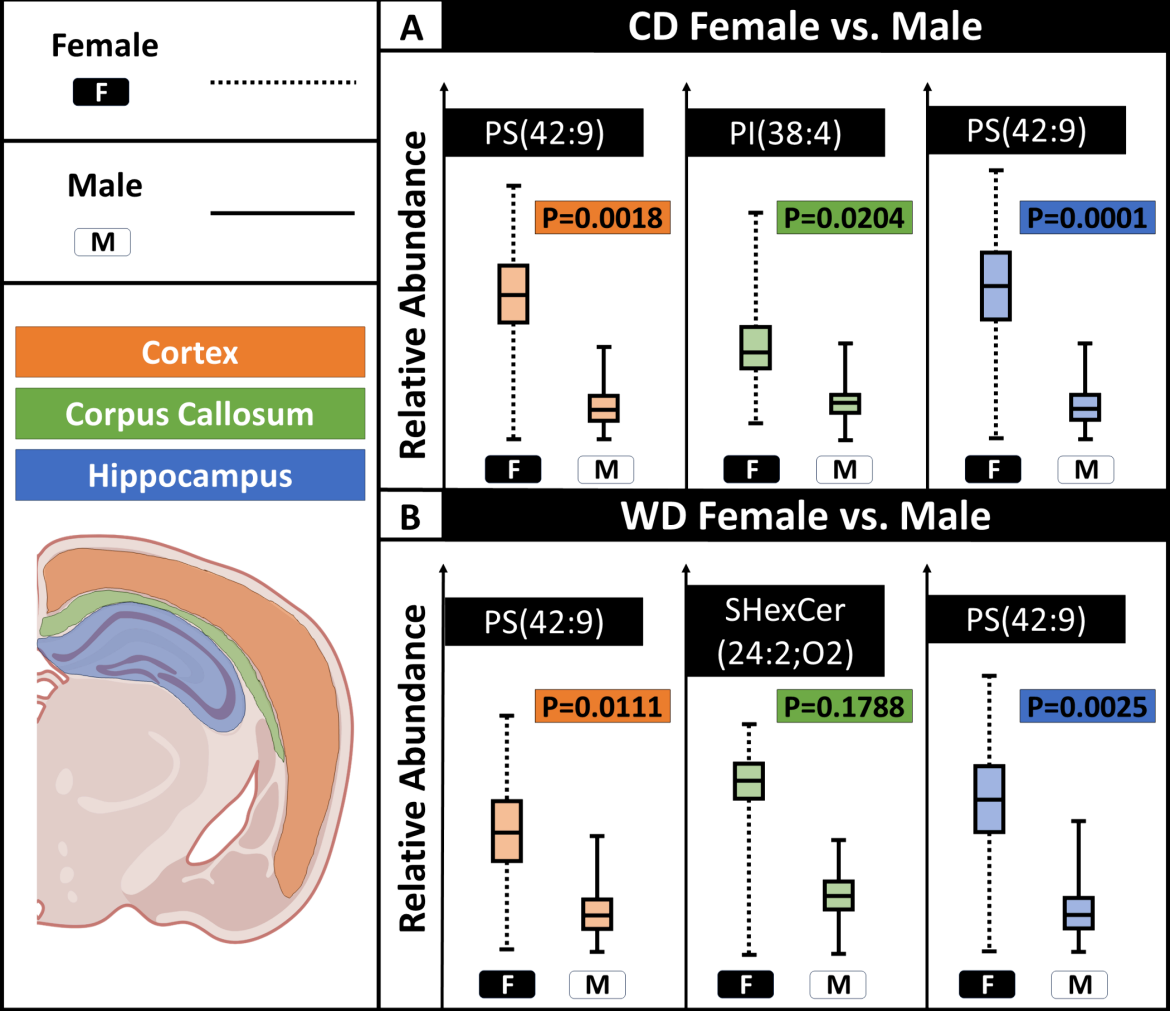
Lipid abundances as a function of sex for different brain regions. (A) For mice fed CD, PS(42:9), PI(38:4), and PS(42:9) were the lipids with highest, significant variation in the cortex, corpus callosum, and hippocampus, respectively. (B) For mice given a western diet, PS(42:9), SHexCer(42:2;O2), and PS(42:9) were the lipids with the highest variation in the cortex, corpus callosum, and hippocampus, respectively. Only SHexCer(42:2;O2) did not show a statistical significance. *N* = 3 male, 3 female per diet (total of six mice).

Characterizing lipids within the brain of mouse models fed WD has been previously performed using GC-MS and LC-MS,^5^ which requires sample homogenization, and thus, loss of all spatial context. Spatial localization of each lipid enables deeper sample characterization by incorporation of segmented brain regions. Because our method allowed for *in situ* lipid assessment in different brain region, we further characterized the lipidomic profile as a function of diet for the cortex, corpus callosum, and hippocampus (Figures 4 and 5). Using the same tissue as above, we performed a comparative analysis in the corpus callosum, cortex, and hippocampus to understand how each region varied with sex and diet. In sum, there are region specific lipid differences that are more dramatic than when considering the whole section (Figures 4 and 5). For comparison of CD and WD fed female mice, HexCer(40:1;O3) and PE(34:0) have the highest ROC scores of 0.73 and 0.72, respectively, within the cortex and corpus callosum. These changes are also statistically significant (*p* value = 0.0119, 0.0450, respectively, Figure 4a). In contrast, lipids localizing to the hippocampus are not statistically significant and have low ROC scores, such as SHexCer(38:6;O5). The cortex has a total of ten lipids with ROC scores greater than 0.7, and 32 lipids with a score greater than 0.6. PE(34:0) is the only detected lipid within the corpus callosum that has a score greater than 0.7, but there are 33 additional lipids having a score greater than 0.6. This demonstrates that both the cortex and corpus callosum have altered lipid profiles in female mice following WD treatment although most lipids have little to no change.

Male mice show slight lipid variation in the cortex and corpus callosum compared to their female counterparts. PE(40:6) and PE(36:1) show the largest changes within the cortex and corpus callosum, respectively, but neither is statistically significant. In total, four lipids in the cortex of male samples have a ROC score greater than 0.6, but none of those exceeds 0.65. Eight lipids within the corpus callosum have a ROC score greater than 0.6, but none exceeded 0.67. PI(38:5) has the highest variation in the hippocampus; however, the ROC score is below 0.6, and the variation is statistically insignificant. Interestingly, lipids with the highest variation in each region after WD treatment were not statistically significant for the male samples. Female mice had significant lipid variation within the cortex and the corpus colosseum between the CD and WD groups. Overall, the female samples showed greater variation than the male samples in the cortex and corpus callosum. Because the baseline lipid profile of the male and female mice samples differed so significantly, it is reasonable to conclude that they would inherently have different lipidomic responses to the WD treatment, as well as other variables not tested here. However, in the hippocampus the male and female samples showed surprising similarity in and lacked lipidomic variation except for PI lipids, which are related to signaling (Table 2). This perhaps demonstrates that the hippocampus is resistant to lipidomic variations within the diet, except for key signaling molecules. However, these mice were only fed WD for 7 weeks; it remains possible that dietary changes over a longer period may be required to affect lipid composition within the hippocampus, especially when compared to the cortex or corpus callosum. However, this does not necessarily infer that the function of the hippocampus is unaffected by the WD, as seen in previous literature and PI lipids. A prior study used LC-MS to analyze lipidomic variation within the hippocampus as a function of a diet rich in saturated fat which showed similarly that the lipid classes generally had no statistically significant variation with a small number of exceptions.^36^ While the diets were different in length and content, these studies both provide evidence that lipids within the hippocampus are difficult to modulate. Alternatively, the cortex and corpus callosum could have altered lipidomic profiles as either a protective function of WD or a negative consequence of WD with adverse effects. Further studies, including behavioral models and aging models, would be needed to determine the functional effects of these variations in the lipidomic profile.

ROC scores show higher lipidomic variation by sex than that by diet (Figure 5). The same regions used for comparing control and WD samples were used to compare the male and female mice. The variation that arose when comparing male and female were significantly higher than for the control vs WD, confirming that there are inherently large variations in the male and female mice that could account for variations in their responses to the WD and differential response to disease.

For CD treated samples, PS(42:9), PI(38:4), and PS(42:9) had the highest upregulation within female mice in the cortex, corpus callosum, and hippocampus, respectively (Figure 5A). For WD treated samples, PS(42:9) has the highest ROC score and is significantly higher in female mice within the cortex and hippocampus. SHexCer(42:2;O2) has the highest variation as a function of sex within the corpus callosum but is not significantly different (Figure 5B). This suggests that PS(42:9) could be more related to sex, while SHexCer(42:2;O2) may be associated with both sex and diet while lacking region specific variation, since it was significantly different when considering the entire section. The cortex and hippocampus shared PS(42:9) as the lipid, with the largest ROC score as a function of sex for both diets. Furthermore, sex-linked lipids show greater statistical significance than those found to vary by diet.

**Figure 6:**
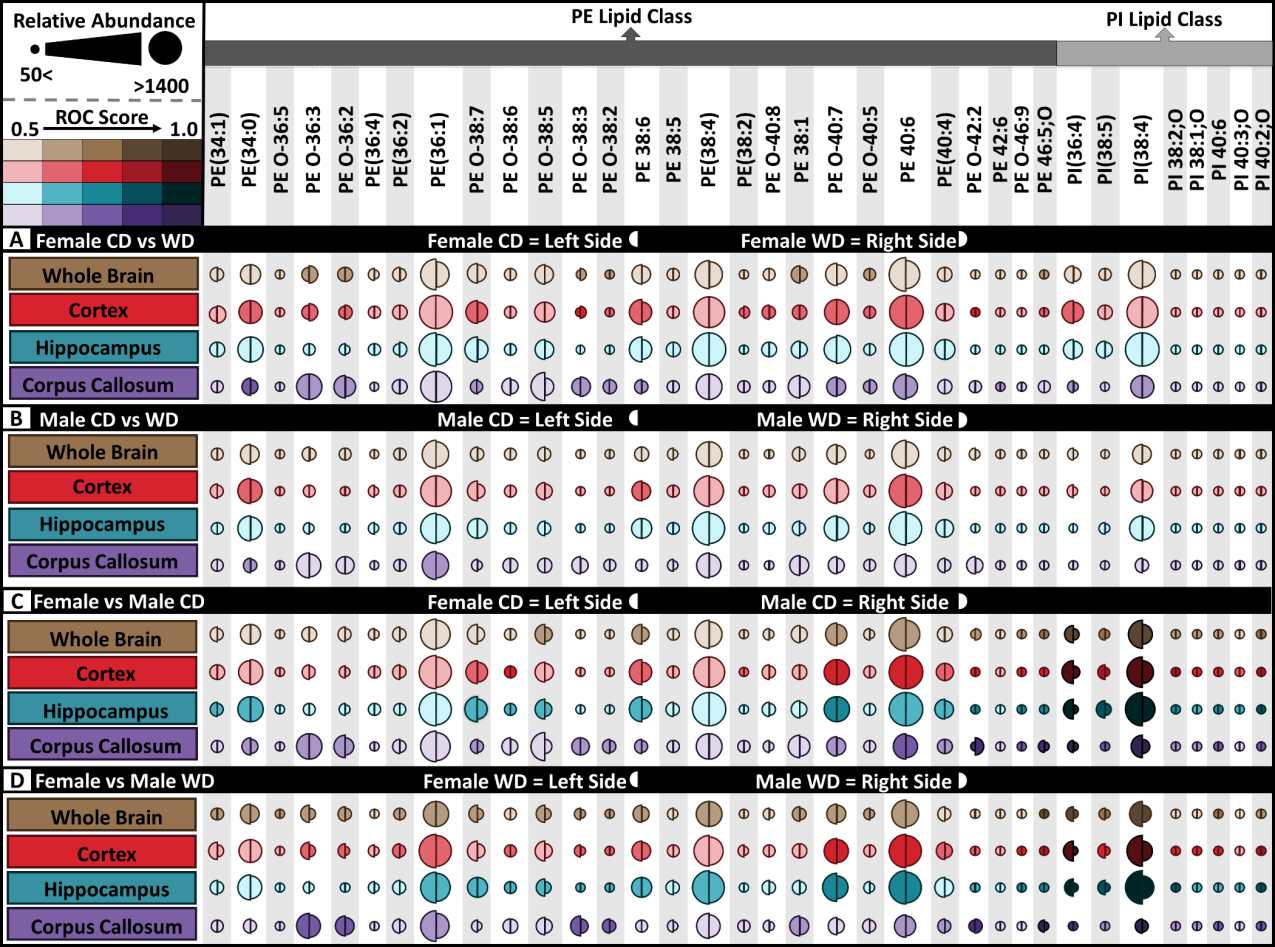
Relative signal intensity and ROC scores for PE and PI lipids within all comparisons. Relative lipid abundance for (A) female CD vs WD (left half-circle = CD, right half circles = WD), (B) male CD vs WD (left half-circle = CD, right half circles = WD), (C) female CD vs male CD (left half-circle = female, right half circles = male), and (D) female WD vs male WD (left half-circle = female, right half circles = male). ROC score increases with color intensity. Darkened color shade represents increased ROC score, and increasing circle radius represents higher lipid ion signal. *N* = 3 male, 3 female per diet.

Out of the 83 putatively identified lipids, 35 are PE and PI lipids, which are structurally similar enough to presumably have similar ionization efficiencies, making it possible to compare lipid abundance between groups. The variance of these lipids as a function of sex and diet are crucial because of the functional importance of the PE and PI lipid groups in cell membranes (Figure 6). PE(40:6) is approximately twice as abundant as PE(38:1) in all regions except for the corpus callosum (Figure 6). The hippocampus is the only region that did not possess lipids with ROC scores greater than 0.6 for male or female mice as a function of diet (Figure 6 A,B). Additionally, the PI lipid class accounted for higher lipidomic variation as a function of diet than did PE lipids (Figure 6C,D). Inversely, PE lipids showed more variation as a function of sex than PI lipids (Figure 6A,B). Overall female mice have higher lipid abundance relative to males regardless of diet, and PE lipid variations appeared more affected by WD in female rather than male mice. One of the most significantly affected lipids based on sex is PI (38:4) which is, significantly higher in female than male regardless of diet (Figures 3D and 6C,D). In general, there is more variation when comparing the sexes than WD and CD, albeit both variables induced lipid changes. The overview of lipids within the whole brain then indicates that a WD could affect the male and female mice differently, contextualizing the importance of sex-matched studies. Additionally, the health ramifications from diet for both male and female mice may be different, as each lipid has a unique function. For instance, PI lipids play an important role in cellular signaling as well as membrane trafficking. For instance, PI isoforms are involved in key signaling enzymatic activities such as phospholipase C (PLC) and phosphoinositide 3-kinase (PI3K).^37,38^ PLC and PI3K are associated with the function of female hormone, estradiol, and its receptor, estradiol receptors (ERs) which are expressed all over the brain, especially in cerebral cortex and hippocampus.^39^ A prior study has shown estradiol-ER activates kinase cascades (ex. PLC/PKC, PI3K/Akt),^40^ crucially involved in synaptic functions that include learning and memory.^41^ ROC scores were higher as a function of sex than diet (Table 2, Figure 6). This is not surprising as studies on WD mice have found that sex and age were greater causes of metabolomic changes than diet, emphasizing that sex and age differences are crucial to account for in diet studies.^13,36^ However, lipidomic variation as a function of sex was detected, especially in individual brain regions. This is also not surprising since WD has been shown to cause inflammation, including in the brain, leading to cognitive decline.^42^ Interestingly, PE lipids showed more abundant changes than PI lipids, perhaps indicating a cellular membrane response as opposed to changes in cellular signaling.

## Conclusion

Diet has a direct effect on human health, but the specific effects at the molecular level in the brain and other parts of the body are not fully understood. WD specifically resulted in changes in the lipidomic profile of the brain and has been linked to neurodegenerative behaviors in mouse models. Our study demonstrated that some lipids varied as a function of sex with fewer varying as a function of diet, with the variance being low in most cases for the whole brain. However, segmenting out specific regions illuminated that there are region-specific lipidomic variations as a function of the sex and diet. Interestingly, female mice showed some variation as a function of diet, while we detected none for male mice. Of the three brain regions analyzed, the cortex is the most varied followed by the corpus callosum. The hippocampus showed a surprising lack of variance as a function of diet, even in cases where some change was observed for the whole brain. Overall, sex had a significantly greater effect on lipidomic differences than diet. However, performing this analysis on mice that have been fed WD for a longer period could elucidate greater lipidomic changes than was observed here. Nevertheless, the significant variation of the lipidome as a function of sex emphasizes the importance of sex-matched studies for neurodegenerative research, especially those that disparately affect one sex. Future experiments include extended periods on altered diets as well as incorporation of orthogonal measurements, such as immunohistochemistry.

## Materials and Methods

### Chemicals and Purification

Chemicals were purchased from Thermo Fisher without further purification, unless otherwise specified.

### Mouse Studies

All protocols involving mouse models were approved by the Institutional Animal Care and Use Committee of the University of California Davis. C57BL/6 wild-type (WT) mice were originally purchased from Jackson Laboratory (Sacramento, CA, USA). At 9 months old, mice were single housed with a 12-h light/dark cycle at 22 °C. Mice were randomly assigned to a treatment group and fed with either a control diet (CD) containing 5.2% fat, 61.3% carbohydrate, and 17.3% protein (w/w, TD. 08485) or a western diet (WD) constituting 21.2% fat, 48.5% carbohydrate, and 17.3% protein (w/w, TD. 88137, Envigo, Indianapolis, IN, USA) for 7 weeks.

### Tissue Preparation

A total of 12 sections were analyzed, including three mice per diet for both male and female cohorts. Whole brains were sectioned to 10 μm on a cryostat (Leica Biosystems, Wetzlar, Germany) at −20 °C. The tissue sections were thaw mounted onto indium tin oxide coated glass slides (Delta Technologies, Limited, Loveland, Colorado).

### Mass Spectrometry Analysis

Tissues were sprayed with 20 mg/mL 1,5 diaminonaphthalene (Tokyo Chemical Industry Co., Tokyo, Japan) in tetrahydrofuran using an HTX TM M3 Sprayer (HTX technologies, LLC, Chapel Hill, NC). Important parameters include flow rate of 0.05 mL/min, 1350 mm/min, 5 total passes, 2 mm track spacing, 40 mm nozzle height, and a nozzle temperature of 40 °C. The samples were run on a Bruker MALDI timsTOF fleX mass spectrometry system (Bruker Scientific, Billerica, MA) immediately after being sprayed. A total of three mice were used within each group (n = 3). The MALDI MSI in negative mode included 150 laser shots, 60% laser energy with the global attenuator set to 100%, 10 μm spatial resolution for 1 sample in each group, and 30 μm for remaining 2 samples in each group resulting in 250,000+ pixels per group. MS method parameters were tuned for detection of lipids (Table 3).

### Data Analysis

Spectra were obtained by total ion count normalized in SCiLS (Bruker Scientific). After normalization, brain regions were manually traced (SI image of the brain regions). Receiver operating characteristics (ROC) plots between groups were used to find lipids of interest. The ROC values were found for the female control vs female WD, male control vs male WD, female control vs male control, and female WD vs male WD comparative groups for the whole brain, cortex, corpus callosum, and hippocampus. P values were calculated using a paired *t* test with N = 3 for each test. Three samples were imaged per group resulting in three average mass spectra. The peak area for each lipid from the average mass spectra of each sample was used for statistical analysis. In cases where subregions were analyzed, SCiLS software was used for segmentation and automatic generation of an average spectrum for each individual region, which was used for statistical analysis as above with the average peak area for each lipid of each sample. Lipids were putatively identified using a combination of mass accuracy (<5 ppm) and LipidMAPS database searching.^43^

## Supporting Information Available

Detected lipid and putative identifications; ROC scores for whole brain, cortex, corpus collosum, and hippocampus; Statistical significance for top five whole brain ROC scor (XLSX)
MALDI MS images for five representative lipids showing female replicates used in study; MALDI MS Images for five representative lipids showing female replicates used in study; method parameters for detection of lipids in negative ion mode using the matrix DAN on a Bruker MALDI timsTOF *flex* (PDF)
