# Supplementary material for: Effects of Sex and Western Diet on Spatial Lipidomic Profiles for the Hippocampus, Cortex, and Corpus Callosum in Mice Using MALDI MSI
Source: J Am Soc Mass Spectrom. 2024 Mar 8;35(11):2554–63. doi: 10.1021/jasms.3c00446 (PMC11544704; doi:10.1021/jasms.3c00446)
Supplement: Supplementary file 2 — js3c00446_si_002.pdf [file js3c00446_si_002.pdf]

Supporting Information.

Effects of sex and western diet on spatial lipidomic profiles of the hippocampus, cortex, and corpus callosum in mice using MALDI MSI.

Catelynn C. Shafer,[1] Jacopo Di Lucente,[2] Ulises Ruiz Mendiola, [2] Izumi Maezawa,[2] Lee-Way Jin,[2] Elizabeth K. Neumann[1*]

Author affiliations

1. Department of Chemistry, University of California, Davis. Davis, CA, 95616
2. Department of Pathology and Laboratory Medicine, University of California Davis, Sacramento, CA95817

*Corresponding author

Corresponding author email: ekneumann@ucdavis.edu

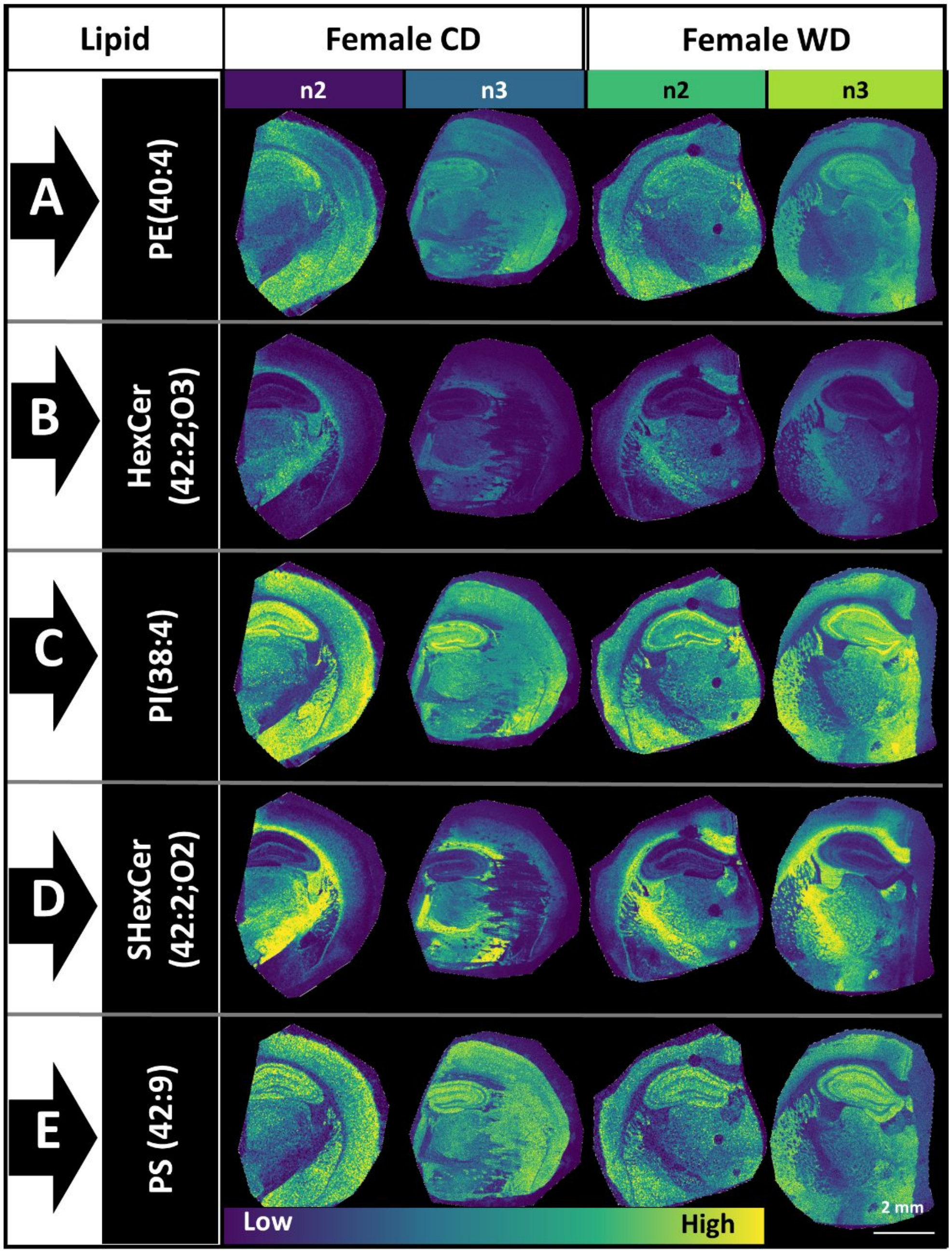

Figure S1: MALDI MS Images for A) PE(40:4) B) HexCer (42:2;O3) C) PI (38:4) D) SHexCer (42:2;O2) and E) PS (42:9) showing female replicates used in study. Note: Areas of tissue damage were excluded from regions.

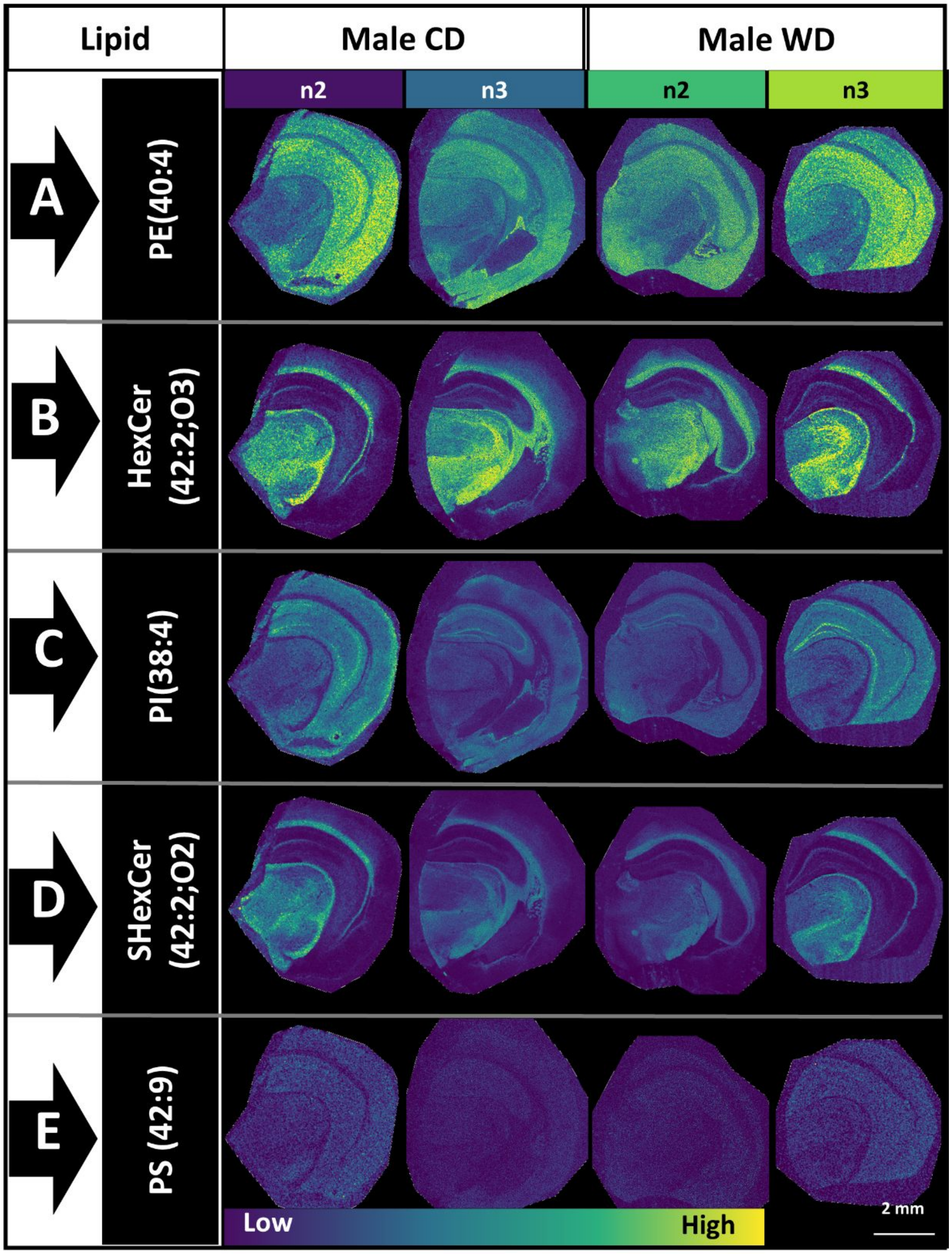

Figure S2: MALDI MS Images for A) PE(40:4) B) HexCer (42:2;O3) C) PI (38:4) D) SHexCer (42:2;O2) and E) PS (42:9) showing male replicates used in study. Note: Areas of tissue damage were excluded from regions.

Table S1 (Excell file): Detected lipid and putative identifications

Table S2 (Excell file): ROC scores for whole brain, cortex, corpus collosum, and hippocampus

Table S3 (Excell file): Statistical significance for top five whole brain ROC scores

Table S4: Method parameters for detection of lipids in negative ion mode using the matrix DAN on a Bruker MALDI timsTOF *flex.*

**Transfer cell**	
MALDI Plate Offset	30.0 V
Deflection 1 Delta	110.0V
Funnel 1 RF	400.0 Vpp
isCID Enerfy	0.0 eV
Funnel 2 RF	400.0 Vpp
Multipole RF	400.0 Vpp
**Collision Cell**	
Collision Energy	5 eV
Collision RF	500.0 Vpp
**Quadrupole**	
Ion Energy	5.0 eV
Low Mass	500.0 m/z
**Focus Pre TOF**	
Transfer Time	80.0 µs
Pre-Pulse Storage	8.0 µs
